# Genetic Variation in Host-Specific Competitiveness of the Symbiont *Rhizobium leguminosarum* Symbiovar *viciae*

**DOI:** 10.3389/fpls.2021.719987

**Published:** 2021-09-08

**Authors:** Stéphane Boivin, Frederic Mahé, Frédéric Debellé, Marjorie Pervent, Mathilde Tancelin, Marc Tauzin, Jerzy Wielbo, Sylvie Mazurier, Peter Young, Marc Lepetit

**Affiliations:** ^1^Laboratoire des Symbioses Tropicales et Méditerranéennes, INRAE, IRD, CIRAD, Montpellier SupAgro, University of Montpellier, Montpellier, France; ^2^Biologie et Génétique des Interactions Plante-Parasite, CIRAD, INRAE, Montpellier SupAgro, University of Montpellier, Montpellier, France; ^3^Laboratoire des Interactions Plantes-Microorganismes, INRAE, CNRS, University of Toulouse, Castanet-Tolosan, France; ^4^Department of Genetics and Microbiology, Maria Curie-Skłodowska University, Lublin, Poland; ^5^Agroecology, AgroSup Dijon, INRAE, University Burgundy Franche-Comté, Dijon, France; ^6^Department of Biology, University of York, York, United Kingdom; ^7^Institut Sophia Agrobiotech, INRAE, CNRS, Côte d’Azur University, Sophia-Antipolis, France

**Keywords:** *Rhizobium leguminosarum* symbiovar *viciae*, competitiveness, pea, fababean, lentil, Fabeae, DNA metabarcoding, symbiosis

## Abstract

Legumes of the Fabeae tribe form nitrogen-fixing root nodules resulting from symbiotic interaction with the soil bacteria *Rhizobium leguminosarum* symbiovar *viciae* (*Rlv*). These bacteria are all potential symbionts of the Fabeae hosts but display variable partner choice when co-inoculated in mixture. Because partner choice and symbiotic nitrogen fixation mostly behave as genetically independent traits, the efficiency of symbiosis is often suboptimal when Fabeae legumes are exposed to natural *Rlv* populations present in soil. A core collection of 32 *Rlv* bacteria was constituted based on the genomic comparison of a collection of 121 genome sequences, representative of known worldwide diversity of *Rlv*. A variable part of the *nodD* gene sequence was used as a DNA barcode to discriminate and quantify each of the 32 bacteria in mixture. This core collection was co-inoculated on a panel of nine genetically diverse *Pisum sativum*, *Vicia faba*, and *Lens culinaris* genotypes. We estimated the relative Early Partner Choice (EPC) of the bacteria with the Fabeae hosts by DNA metabarcoding on the nodulated root systems. Comparative genomic analyses within the bacterial core collection identified molecular markers associated with host-dependent symbiotic partner choice. The results revealed emergent properties of rhizobial populations. They pave the way to identify genes related to important symbiotic traits operating at this level.

## Introduction

Legumes can escape nitrogen-deficit conditions by interacting with rhizobia to form nitrogen-fixing root nodules. In these symbiotic organs, the plant accommodates high densities of differentiated bacteroids able to fix atmospheric N_2_ into ammonium to fuel plant nitrogen metabolism. Natural populations of rhizobia are often genetically diverse (Young, 1985; Young et al., 2021). Bacteria sharing equivalent host-specificity are gathered into symbiovars (sv; Rogel et al., 2011). Genetic diversity based on genomic variation and host specificity are not completely correlated, and sv may include bacteria of multiple species sharing common symbiotic abilities. *Rhizobium leguminosarum* sv *viciae* (*Rlv*) is the specific symbiont of the Fabeae tribe including important crops such as pea (*Pisum sativum* L.), fababean (*Vicia faba* L.), and lentil (*Lens culinaris* L.). The sv *viciae* bacteria generally belong to the *Rhizobium leguminosarum* species complex (*Rlc*) that includes other svs, namely *trifolii* and *phaseoli*. *Rlc* bacteria generally have 4–10 plasmids, one of which carries the main nodulation genes (*nod*) required for symbiosis. Our knowledge of *Rlc* genomic diversity has recently been improved by the release of diverse genome sequences in GenBank (Boivin et al., 2020; Cavassim et al., 2020). *Rlc* is composed of at least 18 different genospecies which are not symbiovar-specific, as bacteria belonging to the same genospecies may display different host-specificities and symbiotic capacities (Kumar et al., 2015; Boivin et al., 2020; Cavassim et al., 2020; Young et al., 2021).

The host specificity of symbiosis is generally the result of Early Partner Choice (Younginger and Friesen, 2019; Walker et al., 2020). EPC is associated with the diversity of the horizontally transferred symbiosis-related regions of the genome, present on specific plasmids or islands (Young, 2016). These regions include the nod genes involved in the synthesis and secretion of lipo-chito-oligosaccharide Nod Factors (NFs) recognized by the plant. The *nodD* gene sequence encoding the transcriptional regulator of the bacterial symbiosis genes, activated in response to plant flavonoids, has been successfully used as a marker to discriminate *Rl* symbiovars (Zézé et al., 2001; Laguerre et al., 2003; Boivin et al., 2020). EPC is usually estimated by the Ability of a bacterium to Form root Nodules with a specific legume host (AFN). However, AFN is not sufficient to predict EPC in natural conditions, when multiple compatible partners interact with the same host. Although most of the *Rlv* bacteria have the capacity to form root nodules with most of the Fabeae legumes, competition occurs between bacteria when they are in populations. Only strains with the highest Competitiveness to Form root Nodules (CFN) finally dominate the symbiotic root system (Ji et al., 2017; Irisarri et al., 2019; Boivin and Lepetit, 2020; Mendoza-Suárez et al., 2020). Therefore, the proportion of the different genotypes in *Rlv* populations associated with Fabeae root nodules do not necessarily reflect the diversity present in soil. *Rlv* CFN varies greatly depending upon the legume host and the *nod* alleles of the bacterial symbiont (Boivin et al., 2020). Moreover, a poor association was often found between CFN and the level of Symbiotic N_2_ Fixation (SNF; Bourion et al., 2017; Boivin and Lepetit, 2020), explaining the frequent failure of inoculation strategies with strains chosen based on high SNF. There is frequently a higher competitiveness of indigenous ineffective bacteria as compared to inoculated strains (Fesenko et al., 1995; Laguerre et al., 2003; McKenzie et al., 2011). AFN and CFN are not the only mechanisms responsible for EPC between symbiotic partners. When the young nodule becomes N_2_-fixing, post-infection mechanisms (Laguerre et al., 2012; Wang et al., 2017) may also modulate the development of the nodules occupied by different bacteria, the differentiation of bacteria in bacteroids, as well as the number of viable bacteria. These mechanisms may result in the “sanction” of inefficient bacteria or may induce the senescence of symbiotic organs (for review, see Boivin and Lepetit, 2020). As symbiotic efficiency of bacteria varies according to its host, it is expected that sanction may depend not only on the microsymbiont but also probably of the plant partner (Regus et al., 2017; Westhoek et al., 2021).

Major mechanisms involved in *Rhizobium*-legume interaction and nodule formation have been elucidated (Oldroyd et al., 2011; Wang et al., 2018). However, very little is known about mechanisms responsible for variation of EPC and CFN. Antibiosis and quorum-sensing mechanisms modulating the multiplication of free-living rhizobia have been reported, and are potentially involved in EPC (Robleto et al., 1997; McAnulla et al., 2007; Naamala et al., 2016). However, even if the preferential proliferation of particular rhizobia within host rhizospheres may contribute to EPC, plant-microbe interactions probably have a major role in EPC (Moawad et al., 1984; Laguerre et al., 2003; Boivin and Lepetit, 2020). Using co-inoculation strategies with high densities of *Rlv* in mixtures, we previously showed that pea and fababean preferentially select some *Rlv* genotypes, although these bacteria have an equivalent capacity to nodulate all partners (Boivin et al., 2020). Some candidate genes and/or genetic markers associated with pea/fababean CFN were identified. Most of these genomic sequences were on plasmids, in agreement with the hypothesis that some horizontally transferred components control CFN. However, the underlying mechanism(s) remain elusive. Candidate genes included nodulation genes (*nod* genes) such as *nodM*, *nodN*, *nodT*, and *nodO*. These genes were previously identified as highly polymorphic (Jorrin and Imperial, 2015). They were suspected to contribute to host specificity (Djordjevic et al., 1985; Surin and Downie, 1988; Lewis-Henderson and Djordjevic, 1991; Baev et al., 1992). Rhizobia produce a large diversity of NFs that bind to legume root LysM-RLK receptors (Oldroyd et al., 2011; Wang et al., 2018). NFs are composed of a chitin-like N-acetyl glucosamine backbone with a fatty acyl chain at the non-reducing end, and carry various substitutions such as glycosylation, acetylation, and/or sulfation on the backbone (Mergaert et al., 1997). NF modifications may influence the binding between NFs and LysM-RLKs and therefore modulate the establishment of the symbiosis and are likely to affect EPC (Dénarié et al., 1992). In agreement with this hypothesis, the *nodX* gene has been reported as involved in a mechanism restricting the AFN and resulting in a specific EPC between *Rlv* bacteria that have *nodX*, such as the strain TOM, and the *Pisum sativum* cultivar “Afghanistan” (Davis et al., 1988). The *nodX* gene encodes an acetyltransferase that modifies NF synthesized by TOM allowing a specific interaction with the LysM-RLK protein encoded within the *SYM2* locus of the “Afghanistan” cultivar (Firmin et al., 1993; Hogg et al., 2002; Sulima et al., 2017). More broadly, the genetic association of *nod* gene diversity with bacterial EPC argues for the hypothesis of an important role of NF in the underlying mechanisms (Boivin et al., 2020). However, other mechanisms, related to plant recognition of bacterial surface polysaccharides or bacterial effectors, have been implicated in the modulation of the legume-*Rhizobium* interaction, and therefore may contribute to EPC (Janczarek et al., 2015; Kawaharada et al., 2015; Miwa and Okazaki, 2017).

In most reported co-inoculation experimental strategies able to reveal CFN variation, bacteria were inoculated with a reference strain or with a limited number of strains (Triplett and Sadowsky, 1992; Laguerre et al., 2003; Bourion et al., 2017). Recently, a co-inoculation strategy with multiple *Ensifer meliloti* strains was applied on two *Medicago truncatula* genotypes illustrating the important impact of the partner choice diversity on several symbiotic traits (Burghardt et al., 2018). However, the phenotyping was done 5 weeks post-inoculation which did not allow to characterize precisely EPC, because post-infection mechanisms likely already modulate EPC. More recently, Mendoza-Suárez et al. (2020), developed a system of DNA tag and reporter genes on plasmids enabling to monitor both competitiveness and N2 fixation in pea inoculated with multiple *Rlv* strains. In this study, taking the new opportunities offered by both NGS and DNA metabarcoding, we designed a strategy to (1) characterize EPC in Fabeae roots inoculated with an artificial *Rlv* population, and (2) identify *Rlv* genes potentially associated with contrasted EPC phenotypes. We defined a core collection representative of the genomic diversity of the *Rlv*. We inoculated this core collection in mixture on three genotypes of each of three Fabeae host species (*Pisum sativum*, *Vicia faba*, and *Lens culinaris*). We used saturating amounts of bacteria to focus on plant-microbe interactions and to reduce impacts of differential bacterial growth. We selected a DNA barcode located on the *nodD* gene to discriminate and quantify individually each *Rhizobium* of the core collection within a nodulated root system using high-throughput NGS. We characterized host-specific EPC profiles of the bacteria forming the core collection. Using a comparative genomic approach between bacterial genomes, we identified *Rlv* genes associated with variation of bacterial EPC in the various plant hosts.

## Materials and Methods

### Bacterial Collection, Inoculation and Plant Growth Conditions

Rhizobia isolated in previous studies by several laboratories from *Pisum sativum, Vicia faba, Lens culinaris*, or *Lathyrus pratensis* root nodules, and from various geographical origins, were used in this study (Supplementary Table 1). The *Pisum sativum* cultivars were “Kayanne”^1^, “Isard”^2^ and “Afghanistan” JI1357^3^. The *Lens culinaris* cultivars were “Rosana,” “Anicia,” and “Flora” (see text footnote 2). The *Vicia faba* cultivars were “Diva,” “Organdi,” (see text footnote 2) and “Tiffany”^4^. Plant seeds were surface sterilized in 3% calcium-hypochlorite solution for 10 min, washed five times in sterilized water, and sown in 2 L pots filled with sterilized perlite/sand (3/1). We inoculated the seeds with the complex inoculum directly after sowing. Bacterial strains were grown individually in YEM broth medium, the number of Colony Forming Units (CFU) was estimated by dilution plating and the identity of each bacterium was systematically checked by PCR amplification and sequencing of the *nodD* gene. Inoculum mixture was obtained by mixing together equal amounts of each bacterium (10^7^ CFU/mL/strain). For each condition, four pots containing four seeds each were used. Plants were grown under high-pressure sodium lamps with a mean photosynthetically active radiation of 250 μmol photons m^–2^.s^–1^ (16/8 h 22/18°C day/night cycle). Plants were irrigated twice a week with N-free HY nutrient solution (KH_2_PO_4_ 1 mM, MgSO_4_ 1 mM, 0.25 mM CaCl_2_, 0.25 mM K_2_SO_4_, 50 μM KCl, 30 μM H_3_BO_3_, 5 μM MnSO_4_, 1 μM ZnSO_4_, 1 μM CuSO_4_, 0.7 μM (NH_4_)_6_Mo_7_O_24_, 100 μM Na-Fe-EDTA adjusted to pH 6.5). Plants were harvested 14 days after inoculation for *Pisum sativum* and *Lens culinaris*, and 21 days after inoculation for *Vicia faba*. Roots were rinsed for 2 min in CaSO_4_ 0,2 mM.

### DNA Extraction, PCR Amplification and Metabarcoding Analysis

We evaluated the proportion of each strain in the symbiotic root nodules by the nod309 DNA metabarcoding strategy. The nodulated root systems of the four plants of each pot were pooled together to form a DNA metabarcoding biological replicate (four biological replicates per condition were generated). We extracted the DNA from whole nodulated root systems ground in liquid N_2_ using the DNeasy Plant Mini Kit^5^. PCR amplifications of the nodD309 barcode sequences were performed using Phusion High-Fidelity DNA Polymerase^6^, specific nodD309 MiSeq primers, and conditions (Supplementary Table 2). We systematically checked the size of the amplicons on agarose gels. Amplicons were sequenced by the Genotoul GeT-PlaGe facility^7^ on an Illumina MiSeq platform using a 2 × 250 bp paired end protocol. Multiplexing was performed using a homemade 6 bp index, which was added to the amplicons during a second PCR with 12 cycles using Miseq_round2 specific primers (Supplementary Table 2). Purified PCR products were loaded onto the Illumina MiSeq cartridge according to the manufacturer’s instructions. Paired Illumina MiSeq reads were assembled with vsearch v2.9.1 (Rognes et al., 2016) using the command fastq_mergepairs and the option fastq_allowmergestagger. Demultiplexing and primer clipping were performed with cutadapt v1.9 (Martin, 2011) forcing a full-length match for sample tags and allowing a 2/3rd-length partial match for forward and reverse primers. Only reads containing both primers were retained. For each trimmed read, the expected error was estimated with vsearch’s command fastq_filter and the option eeout. Each sample was then dereplicated (i.e., strictly identical reads were merged) using vsearch’s command derep_fulllength, and converted to FASTA format. To prepare for clustering, the samples were pooled and processed with another round of dereplication. Files containing expected error estimates were also dereplicated to retain only the lowest expected error for each unique sequence. To detect potential contaminants, the dereplicated data were further clustered with swarm v2.1.9 (Mahé et al., 2015), and checked for chimeras using vsearch’s command uchime_denovo (Edgar et al., 2011). As no significant contamination was detected, downstream analyses, and results are based on unclustered data, only retaining reads strictly identical to the 32 expected *Rhizobium* nodD309 reference sequences (Supplementary Table 3), yielding a total of 511294 reads for all replicates. A mean of 14203 reads per condition was generated (Supplementary Table 4). We calculated the Early Partner Choice (EPC) index of a bacterium in a plant host as the percentage of the nodD309 sequences of the *Rhizobium* of interest divided into the total number of nodD309 sequences generated by all rhizobia of the sample (Supplementary Table 5). EPC index values are the mean of four biological replicates. Variation in the four biological repeats was estimated by calculating the variation coefficient (CV%) for each DNA barcode in all combinations (Supplementary Figure 1). A CV value below 50% was observed for half of the assays indicating an acceptable reliability of the EPC estimate.

### *NodX* Complementation Assays

To investigate if the nodX sequence polymorphism was responsible for nodulation specificity of pea cv “Afghanistan,” 3841 and CCBAU43229, two Nod type B strains lacking nodX, were complemented with the two *nodX* genes from TOM or from P221 rhizobia on transfected plasmids. The strategy was equivalent to Fliegmann et al. (2016) and Sevin-Pujol et al. (2017). Briefly, a broad-host-range cloning vector pFAJ1700GG adapted to the golden gate cloning methodology (Engler et al., 2009) was constructed by inserting a *Sna*BI-*Avr*II fragment from pCAMBIA-CR1 (containing a lacZ cassette; Fliegmann et al., 2016), into *Pme*I-*Xba*I of the pFAJ1700 cloning vector (Dombrecht et al., 2001). In *Rlv*, the *nodX* gene is the last gene of the *nodABCIJX* operon (Davis et al., 1988). A 300 bp DNA fragment carrying a *nod* box located upstream of the *Rlv* TOM *nodA* coding sequence was amplified from genomic DNA using primers abpromnodA1 and abpromnodA2 (Supplementary Table 2), and cloned into pJET vector. The *nodX* coding sequences were PCR amplified from *Rlv* TOM and *Rlv* P221 genomic DNA using primers bdnodxtom1 and bdnodxtom2 (Supplementary Table 2) and cloned into pJET and pGEMT vectors, respectively. The *nod* box containing the promoter and the *nodX* coding sequence modules were assembled in one step into the pFAJ1700GG vector, by a digestion-ligation reaction in the presence of *Bsa*I and T4-DNA ligase as described in Engler et al. (2009). The resulting plasmids, carrying the two versions of *nodX* under the control of the same promoter (DH5a *Escherichia coli* strain), were transferred into the *Rlv* strains 3841 and CCBAU43229 by triparental mating using pRK2013 (in *E. coli* K12) as helper strain (Ditta et al., 1980). For nodulation assays, sterilized pea seeds were germinated on 40/60 v/v pouzzolane/charred clay granules (oil dri from Oil Dri United Kingdom, Bannisters Row, Wisbech, Cambridgeshire) substrate supplemented with Fahraeus medium (Catoira et al., 2000). The growth conditions were 20°C and a mean photosynthetically active radiation of 65 μmol photons m^–2^.s^–1^ (16/8 h 22/18°C day/night cycle). Seedlings were mono-inoculated with 5 ml of a fresh culture (OD_600__nm_ = 0.5) of recombinant rhizobial strains or parental ones. Nodules were scored 3 weeks after inoculation.

### Genome Sequencing, Genomic and Association Genetic Analysis

Bacterial genomes were sequenced by MicrobesNG (Birmingham, United Kingdom)^8^ on an Illumina HiSeq platform using a 2 × 250 bp paired end protocol. Genomic DNA libraries were prepared using Nextera XT Library Prep Kit (Illumina, San Diego, CA, United States). High-quality paired reads were assembled by the Galaxy/BBRIC pipeline^9^ and genome annotations were performed using EuGene-PP (Sallet et al., 2014), and RAST^10^. The pairwise ANI values were calculated using the JSpecies software (Supplementary Table 8)^11^. Strains were assigned to genospecies based on ANI and core gene phylogeny (Young et al., 2021). Heatmaps were built using the pheatmap R package (Kolde, 2019). Presence/absence of genes associated with EPC indexes were identified using the Bidirectional Best Hits (BBHs) tool available in RAST (see text footnote 10). The presence/absence of the specific genes identified was checked by a BLAST search in the genomes of the core collection (thresholds: nucleotide identity >70%; sequence coverage >70%). Kruskal–Wallis tests (eventually followed by a Dunn *post hoc* test with the “Bonferroni” correction for multiple testing) were performed using R software^12^ to test whether the bacterial subgroups displayed equivalent EPC indexes (H_o_ hypothesis), according to diverse criteria (genospecies/Nod types/groups with specific allele). The nucleotide sequences of the *nodABCDEFIJLMN* genes were concatenated and aligned using ClustalOmega^13^, and a Neighbor-Joining (NJ) phylogenetic tree was built using MEGA v7.0.26^14^. The comparisons between reference genomes, and between contigs and the genomic sequence of 3841, for genomic rearrangements/organization (Supplementary Tables 6, 7), were performed using MAUVE software (Darling et al., 2004). Plasmid replicon classes (Rh groups) were identified by sequence similarity of the *repA* gene and presence of cognate *repB* and *repC* (Cavassim et al., 2020).

## Results

### Constitution of a Bacterial Core Collection of Fabeae Rhizobial Symbionts

**FIGURE 1:**
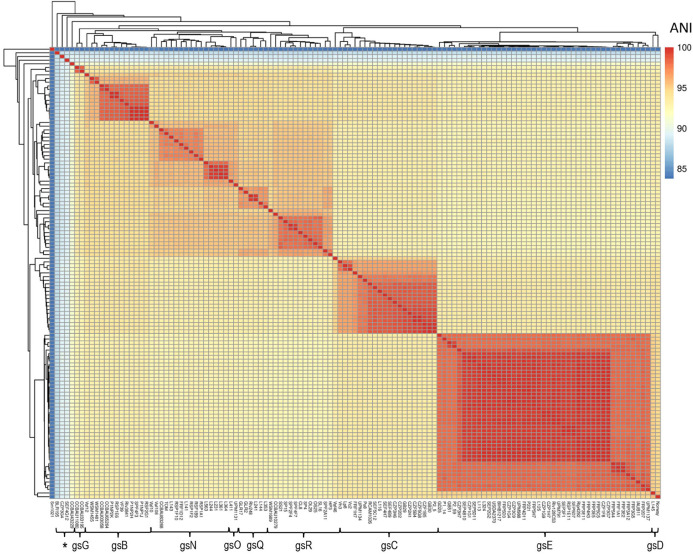
Genomic diversity of the 121 Fabeae symbiont selected for this study. Reference bacteria *Sinorhizobium meliloti* Sm1021, *Rhizobium leguminosarum* sv. *trifolii* WSM1689, *Rhizobium leguminosarum* sv. *phaseoli* Rlp4292 were also included in the comparison. Hierarchical clustering and heatmap were based on the Average Nucleotide Identity (ANI) values between each couple of the rhizobial genomes. *Rlc* genospecies classification (gs) has been based on an ANI threshold of 95%. Star gathered isolates phylogenetically distantly related to *Rlc* (88% < ANI < 92%), but included in the *R. leguminosarum*-*etli* clade. Additional informations are provided in the Supplementary Tables 1, 8.

At the beginning of this study, we collected 73 genome sequences of Fabeae symbionts available in GenBank (Supplementary Table 1). To increase the diversity, the genomes of 48 additional rhizobia from diverse geographical origins, and/or carrying diverse sequences of the symbiotic marker nodD belonging to the symbiovar (sv) viciae, were also sequenced (Supplementary Table 1). All rhizobia have been isolated in previous studies from *Pisum sativum*, *Vicia faba, Lens culinaris*, and *Lathyrus pratensis* root nodules (Supplementary Table 1; Mutch and Young, 2004, Young et al., 2006, Laguerre et al., 2007; Riah et al., 2014; Reeve et al., 2015, Seshadri et al., 2015, Sánchez-Cañizares et al., 2018; Jorrin et al., 2020). Most of the bacteria (117/121) shared an Average Nucleotide Identity (ANI) ¿92% (Figure 1, Supplementary Table 8). They belonged to nine of the *R. leguminosarum* complex (*Rlc*) genospecies previously described: gsB, gsC, gsD, gsE, gsG, gsN, gSO, gsQ, and gsR (Young et al., 2021). Four other strains were phylogenetically distant from the others (88% < ANI < 90%; Supplementary Table 8) and hence outside the *Rlc*, but included inside the *R. leguminosarum-etli* clade according the recent study of Young et al. (2021).

**FIGURE 2:**
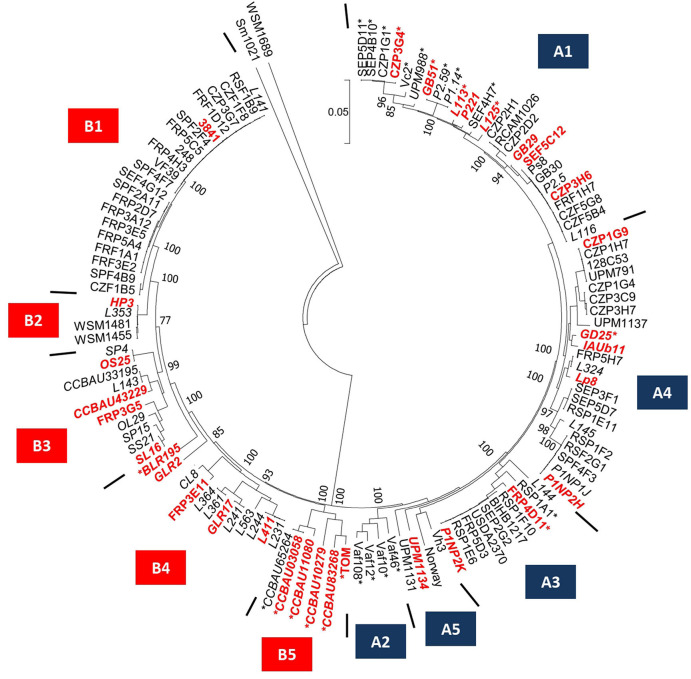
*Nod* gene cluster diversity of the 121 Fabeae symbiont genomes selected for this study. Phylogenetic tree was based on the *nodABCDEFIJLMN* concatenated gene sequences of the 121 *Rlv* genomes. Red and blue boxes defined Nod groups. The 48 new Fabeae symbiont genomes are indicated in italic. The 32 rhizobia of the core collection are indicated in red. Stars indicate rhizobia carrying the *nodX* gene within the *nod* gene cluster. *Rhizobium leguminosarum* symbiovar *trifolii* WSM1689 and *Sinorhizobium meliloti* 1021 have been used as outgroups.

The 121 bacteria of the collection were also discriminated according to their plasmid-borne symbiosis genes. A phylogenetic tree was constructed using the sequence of the 11 conserved *nod* genes located on the symbiosis plasmid (Figure 2). Based on the *nod* gene phylogeny, the *Rlv* bacteria were separated into the two Nod types, named A and B previously described in Boivin et al. (2020). These phylogenetic groups gather strains sharing closely related nod genes sequences and displaying contrasted preferential host specific competitiveness with pea or fababean, respectively (Boivin et al., 2020). Based on the nod genes sequences, these two Nod types were further subdivided into 10 Nod groups named A1–A5 and B1–B5, respectively. In each Nod group, bacteria display *nod* sequence variation, except for the 22 strains of the B1 group that only had very few differences. A total of 44 gs/Nod group combinations were identified within the 121 bacteria, confirming a diversity of association between the symbiosis plasmid and the genospecies (Supplementary Table 6). The number of bacteria per combination is variable. For instance, 19 strains carried the combination gsE/A1 whereas only one strain had gsB/A2 (Supplementary Table 6). The presence of the *nodX* gene within the *nod* gene cluster was surprisingly not restricted to the TOM strain, but was present in 27/121 isolates, including both A and B types (Figure 2).

In order to identify and quantitate individual rhizobial strains in the root system after inoculation with a mixture of many strains, we designed a DNA metabarcoding strategy to evaluate the proportion of each strain in the symbiotic root nodules. We sought a gene that was diverse enough in sequence to act as a natural barcode. The 16S or *gyrB* marker sequences, frequently used in DNA metabarcoding strategies, did not display sufficient intraspecific genetic variation among the 121 strains. Furthermore, they belonged to the chromosome and therefore were poorly genetically linked to most symbiotic phenotypes. The *nodD* gene sequence variations has been already used to discriminate *Rl* bacteria, particularly between and within symbiovars (Zézé et al., 2001; Laguerre et al., 2003; Mutch and Young, 2004; Boivin et al., 2020; Young et al., 2021). We therefore designed a barcode containing a 309 bp fragment of the *nodD* gene, located on the symbiosis plasmid. This sequence displayed 32 *nodD* unique alleles among the 121 strains (Supplementary Table 3). These 32 alleles were distributed among the 10 Nod groups and each Nod group had at least one specific allele (Supplementary Table 6). Based on this barcode, 32 bacteria were finally selected to constitute a Fabeae symbiont core collection, representative of the geographical origins, the plant hosts used for isolation, the diversity of gs/Nod group combinations, and the presence/absence of *nodX* (Table 1, Supplementary Figure 2, and Supplementary Tables 3, 6). Indeed, most geographical origins (87%), all plant hosts used for trapping (100%), and most of the gs/Nod group combinations (67%), were represented in this core collection, which included numerous bacteria with *nodX* (12/32). Nevertheless, it is noteworthy that, because of the limited variation of the barcode, we could introduce only one strain representing the abundant B1 Nod group.

**TABLE 1:** *Rhizobium leguminosarum* symbiovar *viciae* bacteria included in the core collection.

Strains	Isolated from	Locations	Genospecies	Nod groups
3841	*Vicia faba*	United Kingdom	gsB	B1
BLR195	*Lens culinaris*	Bangladesh	*Rhizobium binae*	B4†
CCBAU03058	*Vicia faba*	China	gsB	B5†
CCBAU10279	*Vicia faba*	China	gsR	B5†
CCBAU11080	*Vicia faba*	China	gsG	B5†
CCBAU43229	*Vicia faba*	China	*Rhizobium anhuiense*	B3
CCBAU83268	*Vicia faba*	China	gsN	B5†
CZP1G9	*Pisum sativum*	Czech Republic	gsE	A4
CZP3G4	*Pisum sativum*	Czech Republic	*Rhizobium pisi*	A1†
CZP3H6	*Pisum sativum*	Czech Republic	gsC	A1
FRP3E11	*Pisum sativum*	France	gsE	B4
FRP3G5	*Pisum sativum*	France	gsE	B3
FRP4D11	*Pisum sativum*	France	gsE	A3†
GB29	*Pisum sativum*	Poland	gsC	A1
GB51	*Pisum sativum*	Poland	gsE	A1†
GD25	*Pisum sativum*	Poland	gsE	A4†
GLR17	*Lens culinaris*	Germany	gsQ	B4
GLR2	*Lens culinaris*	Germany	gsQ	B4
HP3	*Pisum sativum*	Algeria	gsR	B2
IAUb11	*Pisum sativum*	France	gsE	A4
L113	*Lens culinaris*	France	gsE	A1†
L125	*Lens culinaris*	France	gsE	A1†
L411	*Lens culinaris*	France	gsO	B4
Lp8	*Lathyrus pratensis*	United Kingdom	gsC	A4
OS25	*Pisum sativum*	Algeria	gsR	B3
P1NP2H	*Pisum sativum*	France	gsB	A4
P1NP2K	*Pisum sativum*	France	gsB	A3
P221	*Pisum sativum*	France	gsE	A1†
SEF5C12	*Vicia faba*	Sweden	gsC	A1
SL16	*Lens culinaris*	Algeria	gsR	B3
TOM	*Pisum sativum*	Turkey	gsN	B5†
UPM1134	*Pisum sativum*	Italy	gsC	A5

*^†^Bacteria carrying the nodX gene within the nod gene cluster.*

### Early Partner Choice Varies Across Fabeae Host Plants

**FIGURE 3:**
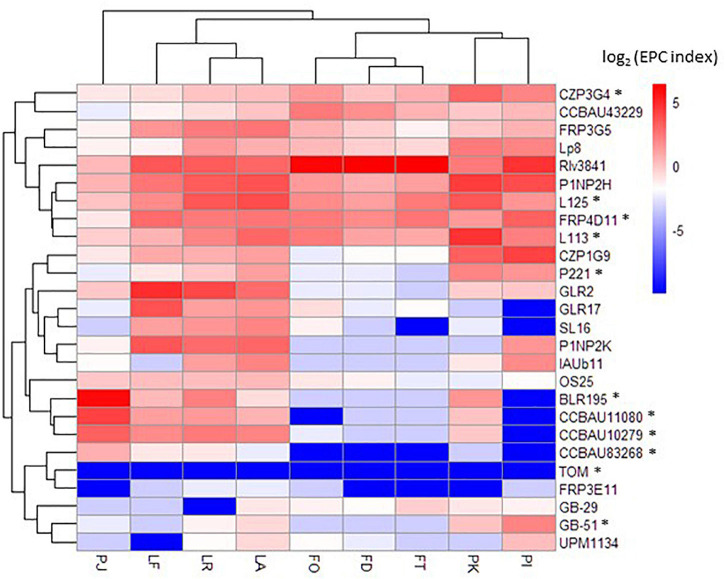
Early partner choice of *Rlv* bacteria with Fabeae plant species/genotypes. Hierarchical clustering and heatmap are based on the EPC indexes for each condition. Only the 20 rhizobia with the best EPC indexes within the core collection are included. Stars indicate the rhizobia carrying the *nodX* gene within the *nod* gene cluster. FO, *Vicia faba* cultivar “Organdi”; FD, *Vicia faba* cultivar “Diva”; FT, *Vicia faba* cultivar “Tiffany”; PK, *Pisum sativum* cultivar “Kayanne”; PI, *Pisum sativum* cultivar “Isard”; PJ, *Pisum sativum* cultivar “Afghanistan J11357”; LF, *Lens culinaris* cultivar “Flora”; LR, *Lens culinaris* cultivar “Rosana”; LA, *Lens culinaris* cultivar “Anicia.”

The 32 *Rlv* bacteria of the core collection constituted the inoculum mixture. We combined saturating densities of each of the 32 bacteria (10^7^ CFU/mL for each strain) to minimize the putative impact of bacterial growth on nodulation success. We inoculated three genotypes of each of the host plant species *Pisum sativum*, *Lens culinaris*, and *Vicia faba*, covering a large genetic diversity of these legume crops, with the *Rlv* core collection. Root systems were harvested once nodules were emerged just starting to be fixating (14 days after inoculation; small and white nodules), and the nodD309 DNA barcode was PCR-amplified from the total DNA extractions. The sequencing of the PCR product yielded numbers of nodD309 sequences, specific to each member of the core collection present in the nodulated root system. Among the 32 bacteria, six were removed from the further analyses as no read was detected in any sample from any host (Supplementary Tables 4, 5). Although the TOM DNA barcode was rare in roots systems of all hosts, few reads were detected and therefore it was included in the analysis. We calculated the Early Partner Choice (EPC) index for each strain in each host plant. The EPC index of the 26 bacteria detected in metabarcoding ranged from 0 to 81.9% (Supplementary Table 5). The mean EPC per bacterium in all hosts ranged from 0.003 to 32.2%. A hierarchical clustering based on the EPC indexes of the 26 bacteria of the core collection detected in metabarcoding separated plant hosts into four groups displaying distinct competitiveness profiles (Figure 3). These four groups were composed, respectively, of the *Vicia faba* genotypes, the *Lens culinaris* genotypes, the *Pisum sativum* cultivars “Kayanne” and “Isard” (cultivated spring and winter peas), and the *Pisum sativum* cultivar “Afghanistan.” For the bacteria the clustering was less marked than for plants (Figure 3 and Supplementary Table 7).

### NodX Is Required but Not Sufficient for Nodulation of *Pisum sativum* cv “Afghanistan”

**FIGURE 4:**
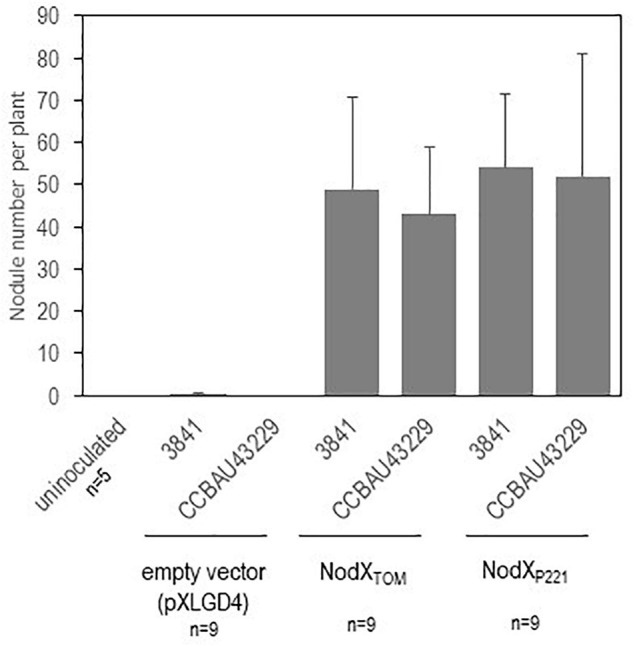
Complementation of Nod type B *Rlv* strains with plasmid constructs expressing NodX. Sterile plantlets of *Pisum sativum* cv. “Afghanistan” were mono-inoculated with various *Rlv* bacteria. 3841 and CCBAU43229 were transformed with empty vector and were compared with the same rhizobial strain expressing NodX from TOM or P221. Nodule number per plant (*n* = 9) were scored 3 weeks after inoculation. Values are mean ± SD.

The *Pisum sativum* cultivar “Afghanistan” (carrying the SYM2 allele) is described in the literature to associate specifically with some *Rlv* strains having the nodX gene (such as the TOM strain that has been frequently used). It is therefore a good case study to validate our metabarcoding strategy. The nodulation profile of this plant genotype was indeed highly divergent compared to the other pea cultivars (Figure 3). Our data confirmed that the bacterial *nodX* gene is required for EPC with this plant genotype, as 89% of the reads detected in nodulated roots of this plant genotype belong to BLR195, CCBAU11080, and CCBAU10279. These three bacteria were not found in such high amounts with other pea cultivars. Nevertheless, neither the presence of *nodX*, nor the Nod type, nor the genospecies, were alone significantly associated with nodulation success on this host (Supplementary Tables 8, 9). In addition to BLR195, CCBAU11080, and CCBAU10279, eight other bacteria of the core collection, belonging to the Nod types A or B, also had the *nodX* gene. However, they did not display a systemically preferential EPC with cv “Afghanistan” (Table 1 and Supplementary Table 9). Only strains having the *nodX* gene and belonging to Nod type B may display high EPC, suggesting that both criteria are required simultaneously for nodulation of pea *cv* “Afghanistan” (Supplementary Table 9). However, this criteria is probably not sufficient as some Nod type B bacteria carrying nodX may display poor EPC with pea *cv* “Afghanistan.” Mono-inoculation experiments with strains carrying *nodX* confirmed that strains with Nod type B were able to form nodules with the pea cv “Afghanistan,” even TOM or CCBAU83868 that showed a poor EPC when inoculated in mixture, whereas the strains with Nod type A were unable, even though they carried *nodX* (Supplementary Table 10). To investigate if the *nodX* sequence polymorphism was responsible for this nodulation specificity, 3841 and CCBAU43229, two Nod type B strains lacking *nodX*, were complemented with the *nodX* gene either from TOM (Type B) or from P221 (type A) rhizobia. All the transformants were able to nodulate the pea cv “Afghanistan” as much as the TOM strain, used as positive control (Figure 4). We concluded that the sequence differences in the NodX protein between the type A and type B strains were not responsible for the inability of the Nod type A strains to form nodules with the pea cv “Afghanistan.” We concluded that factors independent of NodX, present in the Nod type B but absent in Nod type A strains, are necessary for the nodulation specificity with the pea cv “Afghanistan.” Additionally, type B strains carrying nodX, able to form nodules with the pea cv “Afghanistan,” may display different levels of competitiveness to form nodule when inoculated in mixture.

### Host-Specific EPC Factors Are Associated With Nod Type and/or Genospecies

Beyond the particular case of pea cultivar “Afghanistan,” the various Fabeae genotypes form nodules with all the 26 bacteria from the core collection. However, the three other groups of pea, lentil, and fababean cultivars displayed contrasted EPC profiles when inoculated with these bacteria in mixture not reflecting the initial proportion of the complex inoculum (Figure 3). Therefore, this revealed host-specific EPC of the various bacteria. Strain 3841 was particularly predominant in the three *Vicia faba* genotypes (from 70 to 82% of the total reads in the fababean roots) but not in other Fabeae genotypes. This result provided a further validation of the metabarcoding strategy, as 3841 belong to the nod type B1 that was shown to gather strains highly competitive with fababean (Boivin et al., 2020). Unfortunately, as 3841 is the only strain of the core collection belonging to the Nod group B1, it was not possible to extend the comparison to other B1 strains in order, potentially, to associate genetic variation with fababean EPC. However, many strains of the core collection displayed a wide range of EPC variation with the pea and lentil hosts, allowing further investigations. In these cases, it was possible to address the question of the association of these traits with particular genome variants. Strain GLR2 was found at high levels in nodulated roots of the three *Lens culinaris* genotypes, but not in other Fabeae species (Figure 3). In a smaller proportion, contrasted EPC profiles were also observed between plant genotypes of each plant species. For example, strains 3841 and L113 were differentially competitive with the pea cultivars “Kayanne” and “Isard” (Figure 3).

We investigated associations with the Nod type, the Nod group, or the genospecies of the bacteria, at two levels of plant diversity in lentil and pea: the plant cultivar/genotype (Table 2) and the plant species (Table 3). Because of the limited number of strains of the core collection belonging in each category (Nod type, Nod group, and genospecies) and the limited number of plant genotypes, the power of the statistical analysis was limited especially at the cultivar level. Nevertheless, the Nod type was associated with the EPC phenotype in the two pea cultivars “Isard” and “Kayanne,” but not in lentil (Table 2). We identified association with the Nod group with pea cultivard “Isard.” The genospecies was significantly associated with the EPC phenotype in the *Pisum sativum* cultivar “Isard” as well as in lentil cultivars “Anicia” and “Flora.” Investigations at the level of the plant species were obtained by combining the data obtained on the various cultivars (Table 3). Strains with Nod type A generally displayed higher EPC in *Pisum sativum*. Strains of the core collection belonging to the Nod groups A1 and A4 had a higher mean EPC than those belonging to Nod groups B3, B4, and B5 (Table 3). The only exception is 3841, the unique representative of the B1 Nod group of the core collection, which displayed high EPC with pea. This particular result was somehow in contradiction with our previous observations indicating that natural isolates from this Nod group are good competitor with fababean, but not with pea (Boivin et al., 2020). Although EPC indexes of strains belonging to various Nod groups varied widely in lentil, we got no evidence indicating that the Nod type or the Nod group could predict EPC with this plant host. We found associations between genospecies and rhizobial EPC in pea (particularly in the “Isard” cultivar) and in lentil. Some of them are common to both host species. For instance, strains belonging to gsB often displayed high EPC indexes in pea and lentil whereas gsN strains always performed poorly with these hosts. Nevertheless, there were also host-specific examples. For example, strains belonging to gsR were in the medium range of EPC indexes in lentil but in the low range in pea. Despite these global trends, associations were never systematic and exceptions may be observed.

**TABLE 2:** Global effect of the *Rlv* genospecies, Nod types and Nod groups on EPC in particular pea and lentil cultivars inoculated with the *Rlv* core collection.

	Pisum sativum			Lens culinaris		
		
	cv. Kayanne	cv. Isard	cv. Afghanistan	cv. Anicia	cv. Rosana	cv. Flora
Genospecies	n.s	0.0322	n.s	n.s	n.s.	0.0489
Nod type	0.0253	0.0006	n.s	n.s	n.s	n.s
Nod group	n.s.	0.0042	n.s	n.s	n.s	n.s

*Value correspond to the *p*-values of Kruskal Wallis tests, performed to estimate whether significant associations exist between genospecies, Nod types or Nod groups and EPC indexes. The *P*-values are indicated when a significant effect of the genospecies, the Nod type or the Nod group was identified (*p*-value < 0.05). ’n.s.’ not significant.*

**TABLE 3:** Effect of the plant species on the EPC indexes as a function of the *Rlv* Nod types, Nod groups, or genospecies in pea (cv Kayanne and Isard) and lentil (cv Anicia, Rosana and Flora) associated with the *Rlv* core collection.

		Pea			Lentil			pea and lentil	
						
		Median	Dunn pairwise test		Median	Dunn pairwise test		median	Dunn pairwise test
**Nod types**				**Nod types**			**Nod types**		

*p*-value _Kruskal Wallis_			1.90.10^−5^			n.s			4.00.10^−3^
A	n=26	3.95	*a*_4_	n=39	1.9	*–*	n=65	2.9	*a*_6_
B	n=26	0.2	*b*_4_	n=39	2.4	*–*	n=65	1.1	*b*_6_

**Nod groups**				**Nod groups**			**Nod groups**		

p-value _Kruskal Wallis_			4.60.10^−5^			2.70.10^−3^			6.90.10^−6^
A1	n=12	3.45	*a*_1_	n=18	0.75	*b*_2_	n=30	1.9	*b_3_c_3_*
A3	n=4	2.75	*a_1_b_1_*	n=6	7.25	*a*_2_	n=10	6.5	*a_3_b_3_*
A4	n=8	7.15	*a*_1_	n=12	2.25	*a_2_b_2_*	n=20	3.95	*a_3_b_3_*
A5	n=2	0.65	*a_1_b_1_*	n=3	0.3	*b*_2_	n=5	0.3	*c*_3_
B1	n=2	14.45	*a*_1_	n=3	9.9	*a*_2_	n=5	9.9	*a*_3_
B3	n=8	0.6	*b*_1_	n=12	2.6	*a_2_b_2_*	n=20	1.25	*c_3_d_3_*
B4	n=8	0.1	*b*_1_	n=12	2.65	*a_2_b_2_*	n=20	0.85	*c_3_d_3_*
B5	n=8	0	*b*_1_	n=12	0.95	*b*_2_	n=20	0.35	*d*_3_

**Genospecies**				**Genospecies**			**Genospecies**		

*p*-value _Kruskal Wallis_			2.50.10^−3^			1.50.10^−6^			7.10.10^−7^
gsB	*n*=6	9.75	*a*_7_	n=9	9.6	*a*_8_	n=15	9.6	*a*_9_
gsC	*n*=6	0.85	*a_7_b_7_*	n=9	0.4	*b8*	n=15	0.5	*b9*
gsE	*n*=18	3.35	*a_7_b_7_*	n=27	2.3	*ab8*	n=45	2.9	*a_9_ b9*
gsG	*n*=2	0.55	*a_7_b_7_*	n=3	2.3	*ab*_8_	n=5	1.4	*a_9_ b9*
gsN	*n*=4	0	*b7*	n=6	0.1	*b8*	n=10	0	*c9*
gsQ	*n*=4	0.45	*a_7_b_7_*	n=6	10.1	*a*_8_	n=10	2.65	*a_9_ b9*
gsR	*n*=6	0.2	*a_7_b_7_*	n=9	3.2	*ab*_8_	n=15	1.3	*b9*
*R. binae*	*n*=2	1.4	*a_7_b_7_*	n=3	1.3	*ab*_8_	n=5	1.3	*a_9_ b9*
*R. anhuiense*	*n*=2	1.1	*a_7_b_7_*	n=3	0.6	*b8*	n=5	0.9	*a_9_ b9*
*R. pisi*	*n*=2	6.05	*a_7_b_7_*	n=3	1.1	*ab*_8_	n=5	1.2	*a_9_ b9*

*Kruskal Wallis tests were performed to estimate whether bacterial categories were equivalent (Ho hypothesis). P-value are indicated when significant effects of the genospecies, the Nod type or the Nod group on the EPC index were identified (*p*-value threshold= 0,05; ‘n.s’ not significant). *Post-hoc* Dunn pairwise test (with Bonferroni correction for multiple testing) were performed to identify bacterial categories displaying relative differences as indicated by italic letters (a_*i*_, b_*i*_, c_*i*_, d_*i*_).*

### Contrasted EPC Profiles Associate With the Presence of Specific Genes

Comparative genomics analyses identified rhizobial genetic factors associated with the contrasted nodulation profiles. For each plant genotype, we compared the 4–5 bacterial strains with the highest EPC indexes against the 4–5 with the lowest EPC indexes to identify genes specifically present or absent within their genomes. Then, we tested, in the whole set of 26 bacteria that were detected in the root systems, if the presence/absence of the identified genes was significantly associated with the EPC (Supplementary Table 9). Using this strategy, the number of specific genes identified varied from 2 to 13 depending on the plant species and the cultivar. In the particular case of the *Pisum sativum* cv “Afghanistan,” only a fraction the strains of the core collection were able to form nodules with this host. Our first aim was to identify the genetic factors that, together with *nodX*, allow this host-specific nodulation. We restricted the first genomic comparison to the eight bacteria of the core collection carrying the *nodX* gene and belonging either to Nod type A (five strains unable to nodulate this host) or Nod type B (three strains able to nodulate this host). Seventeen genes (named PAFN) associated with ability to form nodule with cv “Afghanistan” were identified by this comparison (Table 4 and Supplementary Table 11). Our second aim was to identify genetic factors specifically associated with competitiveness to form nodule with cv “Afghanistan” by comparing bacteria able to form nodules with this host (i.e., the five Nod type B strains containing the *nodX* gene) but displaying contrasted EPC when inoculated in mixture. The genomic comparisons identified six additional genes (named PA; Table 4 and Supplementary Table 9). We applied a similar strategy for genome comparisons of strains that displayed contrasted EPC in association with pea and lentil (Table 4 and Supplementary Table 9). This analysis yielded a total of 36 candidate genes potentially involved in the EPC of the core collection with pea cultivars “Isard” and “Kayanne” (named PI and PK, respectively) and lentil cultivars “Anicia,” “Rosana,” and “Flora” (named LA, LR, and LF, respectively). Candidate genes involved in many functions such as amino acid transport (L-proline/glycine betaine transporter), amino acid modifications (aminotransferase), nucleic acid repair/modification (DNA/RNA helicase, excinuclease), VapC toxin-antitoxin system, and bacteroid aerotolerance (bat operon), as well as genes with unknown functions were identified (Table 4 and Supplementary Table 14).

**TABLE 4:** Putative functions and replicon location of genes associated with the EPC phenotypes in pea/lentil, identified by the comparative genomic analyses.

			Genomic location					
			
Gene identifier	EPC component	Putative function	3841	Vaf10	Vaf-108	BIHB1217	UPM791	TOM
PK1	CFN	hypothetical protein	NA	Rh08	Rh08	Rh08	Rh08	NA
PK2	CFN	hypothetical protein	NA	NA	Rh02	Rh02	Rh02	NA
PI1	CFN	putative transmembrane	chr	NA	NA	chr	chr	NA
PI2	CFN	putative transmembrane	chr	NA	NA	chr	chr	NA
PI3	CFN	hypothetical protein	chr	chr	NA	chr	chr	NA
PI4	CFN	RelE/StbE replicon stabilization toxin	chr	chr	NA	chr	chr	NA
PI5	CFN	hypothetical protein	chr	NA	NA	chr	chr	NA
PI6	CFN	amidinotransferase	chr	NA	NA	chr	chr	NA
PI7	CFN	amidinotransferase	chr	NA	NA	chr	chr	NA
PI8	CFN	miscellaneous	chr	NA	NA	chr	chr	NA
PI9	CFN	putative transcriptional regulator	Rh03	Rh08	Rh01	Rh08	Rh08	NA
PI10	CFN	hypothetical protein	Rh03	Rh08	Rh01	Rh08	Rh08	NA
PI11	CFN	uracil/thymine DNA glycolase	Rh02	NA	NA	Rh02	Rh02	NA
PI12	CFN	L-2-hydroxyglutarate oxidase	Rh05	NA	NA	Rh04a	Rh04a	NA
PI13	CFN	L-proline/glycine betaine transporter ProP	Rh05	NA	NA	Rh01	Rh01	NA
PA1	CFN	hypothetical protein	NA	chr	chr	NA	NA	NA
PA2	CFN	Delta-9 fatty acid desaturase	NA	chr	NA	NA	NA	NA
PA3	CFN	Putative membrane-bound ClpP-class protease	chr	chr	chr	chr	chr	NA
PA4	CFN	hypothetical protein	Rh02	NA	Rh02	Rh02	Rh02	NA
PA5	CFN	hypothetical protein	chr	chr	chr	Rh01	NA	NA
PA6	CFN	hypothetical protein	NA	Rh08	Rh17	NA	NA	NA
LA1	CFN	TPR domain protein in aerotolerance operon	chr	Rh12	NA	chr	NA	NA
LA2	CFN	BatA	chr	Rh12	Rh01	chr	NA	NA
LA3	CFN	Possible Neuromedin U precursor	chr	Rh12	NA	chr	NA	NA
LA4	CFN	Enoyl-acyl-carrier-protein reductase	chr	NA	NA	NA	NA	NA
LA5	CFN	hypothetical protein	Rh01	Rh01	NA	Rh01	NA	Rh01
LA6	CFN	hypothetical protein	chr	NA	NA	chr	chr	NA
LR1	CFN	Cell filamentation protein fic	chr	Rh08	chr	chr	chr	NA
LR2	CFN	VapC toxin protein antagonist	Rh01	NA	Rh01	NA	NA	Rh01
LF1	CFN	Polyhydroxyalkanoic acid synthase	Rh03	Rh17	Rh17	Rh08	NA	NA
LF2	CFN	Acetyl-coenzyme A synthetase	Rh03	Rh01	NA	Rh08	NA	NA
LF3	CFN	Homoserine O-acetyltransferase	Rh03	Rh01	NA	Rh08	NA	NA
LF4	CFN	putative dehalogenase-hydrolase	Rh03	Rh01	NA	Rh08	NA	NA
LF5	CFN	hypothetical protein	Rh03	Rh01	Rh01	Rh08	NA	NA
LF6	CFN	hypothetical protein	Rh03	Rh01	Rh01	Rh08	NA	NA
LF7	CFN	hypothetical protein	Rh03	Rh01	NA	Rh08	NA	NA
PAFN1	AFN	hypothetical protein	NA	Rh01	Rh01	NA	NA	Rh01
PAFN2	AFN	MFS permease	chr	chr	chr	NA	NA	chr
PAFN3	AFN	hypothetical protein	NA	NA	NA	NA	NA	chr
PAFN4	AFN	Beta-galactosidase	chr	NA	chr	NA	NA	chr
PAFN5	AFN	Beta-galactosidase	chr	NA	chr	NA	NA	chr
PAFN6	AFN	CobN component of cobalt chelatase	chr	chr	NA	NA	NA	chr
PAFN7	AFN	hypothetical protein	NA	Rh12	NA	Rh06	NA	Rh06
PAFN8	AFN	hypothetical protein	Rh12	Rh12	Rh01	Rh06	Rh12	Rh06
PAFN9	AFN	Bll0066 protein	Rh12	Rh12	Rh01	Rh06	Rh12	Rh06
PAFN10	AFN	hypothetical protein	Rh12	Rh12	Rh01	Rh06	Rh12	Rh06
PAFN11	AFN	Probably methylase/helicase	Rh12	Rh12	Rh01	Rh06	NA	Rh06
PAFN12	AFN	nodT RND efflux system	Rh03	Rh01	Rh08	Rh08	NA	Rh06
PAFN13	AFN	Alpha-aspartyl dipeptidase Peptidase E	chr	chr	chr	NA	NA	chr
PAFN14	AFN	putative transmembrane protein	chr	chr	chr	chr	chr	chr
PAFN15	AFN	PE-PGRS FAMILY PROTEIN	chr	chr	NA	NA	NA	chr
PAFN16	AFN	hypothetical protein	chr	chr	NA	NA	NA	chr
PAFN17	AFN	Bll0066 protein	Rh12	Rh12	Rh01	Rh06	Rh12	Rh03

*Replicons are classified (‘chr’ chromosome or ‘rh’ type plasmid) based on sequence homology to repABC genes as described by Cavassim et al., 2020. NA indicates no significant homologous sequence. Gene identifiers referred to the host and the EPC component (CFN or AFN). Gene identified by comparing host-compatible bacteria displaying contrasted level of competitiveness were labelled as CFN and associated to the host used for the comparison: pea cv. ‘Kayanne’ (PK), pea cv. ’Isard’ (PI), pea cv. ‘Afghanistan’ (PA), lentil cv. ‘Anicia’ (LA), lentil cv. ‘Rosana’ (LR) and lentil cv. ‘Flora’ (LF). Gene identified by comparing Rlv strains displaying contrasted ability to form nodule with pea cv ‘Afghanistan’ were labelled AFN (PAFN). More details are provided in Supplementary Table 14.*

Homologs of these genes were sought in the six fully assembled *Rlv* genomes described in GenBank: not only in the reference strain 3841 but also in Vaf10, Vaf108, BIHB1217, UPM791, and TOM. Because *Rlv* genomes contain multiple plasmids actively rearranged, one issue was to define homologous replicons of the various bacteria. The repABC genes control replication and maintenance of plasmids in *Rhizobium*. Their sequence specificities allow replicons to be defined on the basis of the “Rh” putative incompatibility groups (Cavassim et al., 2020). We therefore used “Rh” types to identify analogous replicons in the different bacteria (Table 4 and Supplementary Table 12). We found plenty of evidence for variations and frequent rearrangements between replicons in these genomes. Most of the Vaf10, Vaf108, BIHB1217, UPM791, and TOM plasmids shared homologies with more than one 3841 plasmid, revealing that replicons (and particularly the distribution of sequences among replicons) vary greatly among the various bacteria. For example, in strain BIHB1217, plasmid pPR4 (Rh08) shared sequence homologies with the pRL7 (Rh12), pRL8 (Rh13), and pRL10 (Rh03) plasmids of strain 3841. In the Vaf-108 genome, the chromosome shared homologies with both the 3841 chromosome and the pRL10 plasmid, indicating that rearrangements were probably not restricted to plasmids. This fluidity of the accessory genome was very apparent when we examined the location of the genes that showed significant associations with EPC (Table 4 and Supplementary Tables 13, 14). The genes PI9 and PI10 were always adjacent in the fully assembled genomes: they belonged to Rh01 plasmid in Vaf-108, to Rh03 in 3841, and to Rh08 in Vaf-10, BIHB1217 and UPM791. In the genomes of BIHB1217 and UPM791, the PI12 and PI13 genes belonged to Rh04a, but in 3841 to Rh05. The genes PEPC8-10 and PEPC17 were all on Rh12 in 3841, Vaf-10 and UPM791, on Rh01 in Vaf-108, on Rh06 in BIHB1217. In the strain TOM, PEPC8-10 were on Rh06, but PEPC17 was on Rh03. These multiple examples of genome rearrangement, and numerous others, indicated that the replicon location of a gene could not be reliably inferred from its location in another strain.

## Discussion

This study addressed the question of partner choice at a population level, when multiple compatible host-symbiont associations are possible. In natural soil conditions, the presence of multiple compatible symbiont strains for a given host is generally the rule rather than the exception (Checcucci et al., 2017; Ji et al., 2017; Irisarri et al., 2019; Younginger and Friesen, 2019). In such conditions, the partner choice cannot be simply deduced from the capacities of individual partners but must be analyzed in the context of competition between the various potential associations. As the capacity of symbionts to drive efficient N_2_ fixation activity is variable, this has clearly important consequences for the benefit of symbiosis to the plant. Indeed, several attempts to improve symbiotic traits by inoculation of “elite” rhizobia, selected for their high SNF in individual association with a compatible legume host, have often failed due to outcompetition by endogenous strains already present in the soil (Triplett and Sadowsky, 1992; Fesenko et al., 1995). Improving the knowledge of mechanisms involved in the competitive success of symbiotic association during the EPC stage is necessary to select new “elite” bacteria and design successful inoculation strategies. However, in this study, the plants were cultivated in a standard substrate in controlled laboratory conditions that may differ from a natural ecosystem (which contained generally more complex bacterial populations). Increasing the complexity of the biological system and exploring the impact of soil environment on EPC deserve further investigations.

### A Metabarcoding Approach to Characterize EPC

Previous studies estimated the competitiveness of rhizobial isolates individually by co-inoculating a host plant with both a strain of interest and a reference strain (Bourion et al., 2017; Boivin et al., 2020). Selecting antibiotic resistant mutants, or introducing fluorescent markers on plasmids, greatly improved the recognition of bacteria in these simple mixtures, but could modify the competitiveness of the bacteria of interest (Amarger et al., 1981; Melkonian et al., 2014; Westhoek et al., 2017). Moreover, these techniques hardly consider the effect of multiple interactions. Construction of bacterial populations in which each strain can be individually quantified, allows the study of complex interactions that cannot be revealed when single strains are competed separately with a reference strain (Epstein et al., 2018; Carlström et al., 2019; Boivin and Lepetit, 2020). The use of the DNA metabarcoding strategy allowed EPC to be evaluated after co-inoculation of roots with large populations of diverse unmodified rhizobia. Previous studies have estimated rhizobial EPC by the number of nodules formed with the bacteria of interest as compared to total nodule number on the plant root system. Estimating the EPC by a DNA metabarcoding approach globally quantifies rhizobia interacting with the plant. However, differential nodule growth, or differential levels of bacteroid endoreduplication, of the various bacteria may result in differences between these two estimates (Kazmierczak et al., 2017). To minimize this potential difference, we applied DNA metabarcoding on roots at an early stage of interaction, before these processes become prominent. This metabarcoding strategy was able to confirm and extend previous data already obtained by earlier methods: (1) in pea cv Afghanistan the host specific nodulation of some strains that produce NodX, (2) in fababean the high EPC of Nod type B strains. In both cases, co-inoculation of the hosts by the core collection of strains resulted in the expected EPC profiles, validating our strategy. Our global approach to measure EPC, addressed at the whole root system level, was able to confirm conclusions obtained by earlier time-consuming experimental strategy addressed at the nodule level with a limited number of strains (Boivin et al., 2020).

### A Representative Core Collection of Fabeae Symbionts

Sequencing 48 additional genomes of *Rlv* strains isolated from Fabeae root nodules of various geographical origins allowed us to characterize and extend our current knowledge of the *viciae* symbiovar and genospecies diversity, increasing the 73 *Rlv* genome sequences available in GenBank at the beginning of this study. Genospecies were defined based on established criteria for bacteria in general as well as for *Rhizobium* species (Jain et al., 2018; de Lajudie et al., 2019; Young et al., 2021). All the bacteria shared closely related *nod* gene sequences typical of the symbiovar *viciae*. Their core genes placed the majority of them within genospecies in the *R. leguminosarum* species complex (Young et al., 2021), but several isolates were genetically distant from these (ANI < 90%) and belonged to other species in the wider *R. leguminosarum – R. etli* clade (Supplementary Table 1). We selected a set of 32 bacteria, representative of the known genomic diversity of Fabeae symbionts, to study host-specific EPC. Individuals within the bacterial mixture have been discriminated by a DNA barcode located in the *nodD* gene of the symbiotic plasmid. We assumed that the number of barcodes of each strain was proportional to their abundancy in the nodules of the root system. This *nodD* metabarcoding enabled us to explore much of the intraspecific variability of the symbiovar. Nevertheless, within a few bacterial subgroups, the barcode diversity is limited. Namely, the *Rlv* strains carrying the Nod type B1 shared an identical barcode and only one strain could be included in the EPC study although strains of this subgroup may display genomic variations. The design and the high-throughput sequencing of a longer barcode might improve the resolution of the technique in the near future.

### Inoculation of *Rlv* Core Collection on Various Hosts Revealed Host-Specific EPC

Co-inoculation of the core collection of 32 *Rlv* strains, in mixture, on *Pisum sativum*, *Vicia faba*, and *Lens culinaris* genotypes revealed contrasted host-specific EPC profiles. Profiles varied according to the bacterium, the plant species and the plant genotype. They resulted from the different capacities of the various plant-microbe partnerships. Because bacteria were amplified separately and were present at high density in the inoculum (>10^7^ CFU/mL), these contrasted EPC profiles were unlikely to be due to microbe-microbe interactions resulting in differential bacterial multiplication in the rhizosphere, possibly limiting root infection. This study identified bacteria of the *Rlv* core collection that displayed host-specific EPC with diverse *Vicia faba*, *Pisum sativum*, and *Lens culinaris* genotypes. In a few cases, the partner choice was the result of a specific ability to form nodules that was confined to certain strains (namely Nod type B bacteria with the *nodX* gene and pea cv “Afghanistan”) allowing them to associate with this host while others could not. However, in most case *Rlv* strains have abilities to associate with all the Fabeae hosts but display different levels of EPC when co-inoculated in mixture, resulting in contrasted responses of the various hosts to the co-inoculation with the *Rlv* core collection. This offered the opportunity to associate ability to nodulate and EPC traits of the bacteria with specific genome variations.

### Both the *nodX* Gene and Nod Type B-Related Factors Are Necessary to Confer Ability to Form Nodules With the *Pisum sativum* Cultivar “Afghanistan”

The co-inoculation strategy confirmed earlier data indicating that only *Rlv* bacteria carrying the *nodX* gene can nodulate with the pea cultivar “Afghanistan,” resulting in a highly specific association profile, consistent with a restriction of host specificity in this particular plant genotype (Davis et al., 1988; Firmin et al., 1993). However, this study revealed unexpected complexities of the *nodX*/Sym2 interaction. Surprisingly many *Rlv* genomes (22%), belonging to both A and B *nod* types, have a *nodX* gene. The corresponding bacteria were isolated from different Fabeae host plants and have multiple geographical origins around the world, including locations where relatives of cv “Afghanistan” are not expected to be present. Nodulation tests confirmed that the *nodX* gene from a Nod type A strain (i.e., strain P221) is able to complement a Nod type B strain lacking *nodX* and allow the transconjugant to nodulate cv. “Afghanistan” even though the donor cannot. This rules out the hypothesis of a non-functional copy of *nodX* in Nod type A strains. If the *nodX* function is related to the host-specific nodulation with the pea cv “Afghanistan,” why has the gene been maintained in the Nod type A bacteria? We suggest that other unknown functions might explain the conservation of this gene within natural *Rlv* populations. Interestingly, the *nodX* gene is also present in *R. leguminosarum* symbiovar *trifolii* rhizobia, which nodulate clovers but are unable to nodulate any pea genotype, including cv “Afghanistan” (Ovtsyna et al., 1999). Other surprising results were that (1) the *nodX* gene was not sufficient alone to confer ability form root nodules with the cultivar cv “Afghanistan” to all *Rlv* bacteria, and (2) other genetic determinants specific to the *nod* type B strains were also required together with *nodX*. Therefore, the control of the host-specific nodulation with the cv “Afghanistan” in *Rlv* might be more complex than deduced from earlier studies (Davis et al., 1988; Firmin et al., 1993). Finally, this study showed that the ability to nodulate conferred by both the Nod type B and the presence of *nodX* does not guarantee preferential EPC when multiple compatible partner are co-inoculated. A striking example is the TOM strain, the well-studied reference symbiont of the pea cv “Afghanistan.” This strain, able to form nodule with this cultivar when mono-inoculated, was included in the co-inoculation mixture. However, it was detected at very low level in the symbiotic roots because completely outcompeted by other Nod type B *Rlv* strains that also have the *nodX* gene. This revealed that this subgroup of strains with specific capacities to associate with this host gathered strains with contrasted levels of competitiveness to form nodules.

### Host-Specific EPC Is Associated With Different Genomic Regions of *Rlv* Bacteria

Our metabarcoding strategy on Fabeae plants, inoculated with the population of the *Rlv* core collection, was able to confirm our previous results indicating that host-specific EPC was associated with the *Rlv* Nod types A and B with pea and fababean, respectively (Boivin et al., 2020). Since *nod* genes are directly related to NF production and secretion (Mergaert et al., 1997), the associations between the Nod types/groups and EPC deserves further investigations. The quantity and particularly the quality of the NF cocktail produced may contribute to the level of competitiveness of the bacteria: this is an interesting hypothesis that will need further investigations. We obtained, in this study, new evidence indicating that the EPC with lentil was associated with the genospecies, but poorly with the Nod type or the Nod group, in contrast to the results for pea and fababean. Indeed, different genetic determinants, mainly located on the chromosome, controlled EPC with lentil. This confirms the report of Jorrin and Imperial (2015) suggesting that lentil may be less selective than pea and fababean for rhizobial partner choice. Nevertheless, despite these global trends, the genetic control of EPC was always complex since, for all plant hosts, we found associations of EPC with both Nod groups and genospecies, as well as an interaction. An unexpected result was the observation of a high EPC of the 3841 bacteria with pea. The 3841 strain is the unique bacterium of Nod group B1 present in the core collection. Our previous investigation on natural isolates in comparison with a reference strain indicated that bacteria of this group B1 were generally competitive with fababean but poorly competitive with pea (Boivin et al., 2020). This different behavior of the strain 3841 as compare to natural isolates deserves further investigation with particular emphasis on the association with the pea EPC phenotype. Because the strain 3841, initially isolated from pea root nodules, was cultivated in laboratories for many years, we cannot rule out that this particular phenotype may be related to unknown selection processes. A similar comment could be done with the strain TOM which was also isolated long time ago. It could be speculate that laboratory selection could have produced the opposite effect compare to 3841, and reduced its competitiveness with its original pea host. Nevertheless, the strategy of comparing genomes between competitive and uncompetitive strains allowed us to identify, for each plant genotype, regions of the bacterial genomes associated with host-specific EPC. The assignment of the identified genomic regions to a particular replicon deserves further study. As the knowledge of *R. leguminosarum* genomes is expanding, increasing evidence indicates that plasmid number, size and composition vary greatly in this bacterial species (Mazurier and Laguerre, 1997; Laguerre et al., 2003; Kumar et al., 2015; Cavassim et al., 2020). Different bacteria belonging to the same symbiovar share homologous sequences, organized differently in their diverse replicons. There is evidence that genetic rearrangements may occur even between plasmids and chromosome. The emerging picture revealed a high fluidity between *R. leguminosarum* replicons that argued against our first attempt to assign genomic regions to replicons by homology with a reference genome. More recently, the intensity of the rearrangements and the complexity of the evolution *Rhizobium* plasmids as compared to chromosome, as well as symbiotic clusters as compared to other accessory clusters, have been evidenced using whole genome analysis (Li et al., 2020). However, the fluidity of the accessory genome is essential for the genetic association approach that we have taken in this study, which requires a certain degree of independence between loci in order to associate phenotypes with individual genes rather than with whole replicons or large blocks of co-inherited genes.

### Candidate Genes Involved in Host-Specific EPC

As comparative studies yielded multiple potential genomic regions associated with EPC, it is tempting to hypothesize that multiple genes control this trait. Some protein sequences have functions already associated with EPC. For instance, the rhizobial *nodT* gene, putatively involved in the secretion of nod factor (Rivilla et al., 1995), as well as components of Vap toxin/antitoxin systems (Lipuma et al., 2014), were already suspected to be associated with EPC with pea or fababean in a previous study based on co-inoculation experiments with a reference strain (Boivin et al., 2020). Here, we identified new candidate genes associated with EPC. However, these genetic associations have to be validated as it may be related to neighboring loci. Therefore, further investigations are required to demonstrate the direct role of these genes in EPC. Reverse genetics studies with different combinations of alleles will be required to validate their potential biological function in host-specific EPC of *Rlv* strains. The expression of the candidate genes could reveal differential expression depending on environmental conditions and/or host specificities (Fagorzi et al., 2021). Nevertheless, these sequences are valuable markers to select competitive *Rlv* strains with pea and/or lentil and pave the way for the identification of the *Rlv* genes controlling EPC. This knowledge opens new perspectives to select rhizobia and legumes, by genetic association, for new inoculation strategies that will ultimately improve the agro-ecological services of Fabeae legume holobionts (Burghardt, 2020). Moreover, this metabarcoding strategy coupled with complex inocula could be also be extended to other beneficial or pathogenic plant-microbe interactions.

## Data Availability Statement

New bacterial genomes sequences (48) are described in the BioProject PRJNA579265 that gathered 48 BioSamples (https://www.ncbi.nlm.nih.gov/). Genome accession numbers of these genome sequences are in the Supplementary Table 1, together with the accessions numbers of genomes already described in previous studies included in our analysis. DNA NodD Metabarcoding sequences are described in the BioProject PRJNA744092 that gathered 36 BioSamples (https://www.ncbi.nlm.nih.gov/). The BioProject and the BioSamples are described in the same way as in the article. Each BioSamples has two fastq files due to paired-end sequencing (raw data). The 30 barcodes sequences of the core-collection bacteria are presented in Supplementary Table 3. Numbers of barcodes in biological samples after filtering by the pipeline described in the section “Materials and Methods” of the article is presented in Supplementary Table 4.

## Author Contributions

ML and SB designed the research and wrote the manuscript with the contribution of PY. ML, SB, PY, JW, and SM provided rhizobial strains. SB, FD, MP, MatT, and FM performed the experiments. FM, MarT, and SB performed genomic and metabarcoding *in silico* analysis. SB, ML, FM, FD, and PY analyzed the data. All authors contributed to the article and approved the submitted version.

## Conflict of Interest

The authors declare that the research was conducted in the absence of any commercial or financial relationships that could be construed as a potential conflict of interest.

## Publisher’s Note

All claims expressed in this article are solely those of the authors and do not necessarily represent those of their affiliated organizations, or those of the publisher, the editors and the reviewers. Any product that may be evaluated in this article, or claim that may be made by its manufacturer, is not guaranteed or endorsed by the publisher.

## Footnotes

1
www.kws.com

2
www.agriobtentions.fr

3
https://www.seedstor.ac.uk/

4
www.ragt-semences.fr

5
www.qiagen.com

6
www.thermofisher.com

7
https://get.genotoul.fr

8
https://microbesng.uk/

9
https://bbric-pipelines.toulouse.inra.fr/

10
http://rast.nmpdr.org/

11
http://jspecies.ribohost.com/jspeciesws

12
http://www.R-project.org/

13
www.ebi.ac.uk

14
www.megasoftware.net

## Supplementary Material

Supplementary Figure 1 Variation of the EPC index.

Supplementary Figure 2 NodD309 barcode diversity among *Rhizobium leguminosarum* sv. *viciae.*

Supplementary Table 1 Genomic data of the sequenced *Rlv* bacteria.

Supplementary Table 2 Primers used and PCR conditions for metabarcoding.

Supplementary Table 3 NodD309 sequences of the 32 rhizobia included in the *Rlv* core collection.

Supplementary Table 4 Read numbers in each sample after quality filtering.

Supplementary Table 5 EPC indexes for each of the plant/rhizobia pairs.

Supplementary Table 6 Genospecies and Nod group combinations in the initial rhizobial collection and within the core-collection.

Supplementary Table 7 Clusters of *Rlv* bacteria of the core collection, gathered on the basis of the EPC profiles of the Figure 3.

Supplementary Table 8 Average Nucleotide Identity (ANI) between the 121 genomes of *Rlv*, as well as *Sinorhizobium meliloti* Sm1021, *Rl* sv *trifolii* WSM1689 and *Rl* sv *phaseoli* Rlp4292.

Supplementary Table 9 Presence/absence of genomic regions associated with EPC phenotypes in pea, fababean, and lentil.

Supplementary Table 10 Mono-inoculation of the *Pisum sativum* cv “Afghanistan” with some Nod types A and B NodX-*Rlv* strains, included in the *Rlv* core collection.

Supplementary Table 11 Presence/absence of genomic regions in Nod-type A and Nod-type B *Rlv* strains carrying NodX, associated with an EPC with *Pisum sativum* cv “Afghanistan”.

Supplementary Table 12 Global rearrangements of plasmids/contigs between reference strains, in comparison with 3841.

Supplementary Table 13 Genomic organization of contigs containing genes associated with EPC, compared to 3841.

Supplementary Table 14 Putative functions and locations of genes associated with EPC from the comparative genomic analyses.
