# Corrigendum: Genetic variation in host-specific competitiveness of the symbiont *Rhizobium leguminosarum* Symbiovar *viciae*

**DOI:** 10.3389/fpls.2025.1542763

**Published:** 2025-02-24

**Authors:** Stéphane Boivin, Frederic Mahé, Frédéric Debellé, Marjorie Pervent, Mathilde Tancelin, Marc Tauzin, Jerzy Wielbo, Sylvie Mazurier, Peter Young, Marc Lepetit

**Affiliations:** ^1^ Laboratoire des Symbioses Tropicales et Méditerranéennes, INRAE, IRD, CIRAD, Montpellier SupAgro, University of Montpellier, Montpellier, France; ^2^ Biologie et Génétique des Interactions Plante-Parasite, CIRAD, INRAE, Montpellier SupAgro, University of Montpellier, Montpellier, France; ^3^ Laboratoire des Interactions Plantes-Microorganismes, INRAE, CNRS, University of Toulouse, Castanet-Tolosan, France; ^4^ Department of Genetics and Microbiology, Maria Curie-Skłodowska University, Lublin, Poland; ^5^ Agroecology, AgroSup Dijon, INRAE, University Burgundy Franche-Comté, Dijon, France; ^6^ Department of Biology, University of York, York, United Kingdom; ^7^ Institut Sophia Agrobiotech, INRAE, CNRS, Côte d’Azur University, Sophia-Antipolis, France

**Keywords:** *Rhizobium leguminosarum* symbiovar *viciae*, competitiveness, pea, fababean, lentil, Fabeae, DNA metabarcoding, symbiosis

In the published article Mutch et al., 2004, Young et al., 2006, Laguerre et al., 2007; Riah et al., 2014; Reeve et al., 2015, Seshadri et al., 2015, Sánchez-Cañizares et al., 2018; Jorrin et al., 2020 were not cited in the article. The citation has now been inserted in the **Result** section, first paragraph and should read:

“At the beginning of this study, we collected 73 genome sequences of Fabeae symbionts available in GenBank (Supplementary Table 1). To increase the diversity, the genomes of 48 additional rhizobia from diverse geographical origins, and/or carrying diverse sequences of the symbiotic marker nodD belonging to the symbiovar (sv) viciae, were also sequenced (Supplementary Table 1). All rhizobia have been isolated in previous studies from *Pisum sativum*, *Vicia faba*, *Lens culinaris*, and *Lathyrus pratensis* root nodules (Supplementary Table 1; Mutch et al., 2004, Young et al., 2006, Laguerre et al., 2007; Riah et al., 2014; Reeve et al., 2015, Seshadri et al., 2015, Sánchez-Cañizares et al., 2018; Jorrin et al., 2020). Most of the bacteria (117/121) shared an Average Nucleotide Identity (ANI) *>*92% (Figure 1, Supplementary Table 8). They belonged to nine of the *R. leguminosarum* complex (*Rlc*) genospecies previously described: gsB, gsC, gsD, gsE, gsG, gsN, gSO, gsQ, and gsR (Young et al., 2021). Four other strains were phylogenetically distant from the others (88% *<* ANI *<* 90%; Supplementary Table 8) and hence outside the *Rlc*, but included inside the *R. leguminosarum-etli* clade according the recent study of Young et al. (2021).”.

In the published article, the respective original sources of the newly 48 sequenced Rlv strains were ambiguous.

A correction has been made to **Material and method** section, Bacterial Collection, Inoculation and Plant Growth Conditions subsection, first paragraph, first sentence. This sentence previously stated:

“Rhizobia isolated from Pisum sativum, Vicia faba, Lens culinaris, or Lathyrus pratensis root nodules, and from various geographical origins, were collected (Supplementary Table 1)”

The corrected sentence appears below:

“Rhizobia isolated in previous studies by several laboratories from Pisum sativum, Vicia faba, Lens culinaris, or Lathyrus pratensis root nodules, and from various geographical origins, were used in this study (Supplementary Table 1).”

A correction has been made to **Aknowledgment** section, Bacterial Collection, Inoculation and Plant Growth Conditions subsection, first paragraph, first sentence. This sentence previously stated:

“We acknowledge Markus Braun (Heidelberg University), Xinhua Sui and Changfu Tian (China Agricultural University, Beijing), Anna Skorupska and Andrzej Mazur (Medical University of Lublin), and Juan Imperial (Instituto de Ciencias Agrarias, Madrid) for providing some of the rhizobial strains included in this study, as well as the Genotoul GeT-PlaGe platform of Toulouse for the Illumina MiSeq sequencing.”

The corrected sentence appears below:

“We acknowledge Markus Braun (Heidelberg University), Xinhua Sui and Changfu Tian (China Agricultural University, Beijing), Anna Skorupska and Andrzej Mazur (Medical University of Lublin), Juan Imperial (Instituto de Ciencias Agrarias, Madrid), Nassira Riah (University of Constantine) for providing some of the rhizobial strains included in this study, as well as the Genotoul GeT-PlaGe platform of Toulouse for the Illumina MiSeq sequencing.”

In the published article, there was an error in Supplementary Table 1, the sources of the newly 48 sequenced Rlv strains were not properly detailed.

The authors apologize for these errors and state that this does not change the scientific conclusions of the article in any way. The original article has been updated.
