# Supplementary material for: Genetic Variation in Host-Specific Competitiveness of the Symbiont Rhizobium leguminosarum Symbiovar viciae
Source: Front Plant Sci. 2021 Sep 8;12:719987. doi: 10.3389/fpls.2021.719987 (PMC8457355; doi:10.3389/fpls.2021.719987)
Supplement: Supplementary Figure 1 — Variation of the EPC index. [file Data_Sheet_1.PDF]

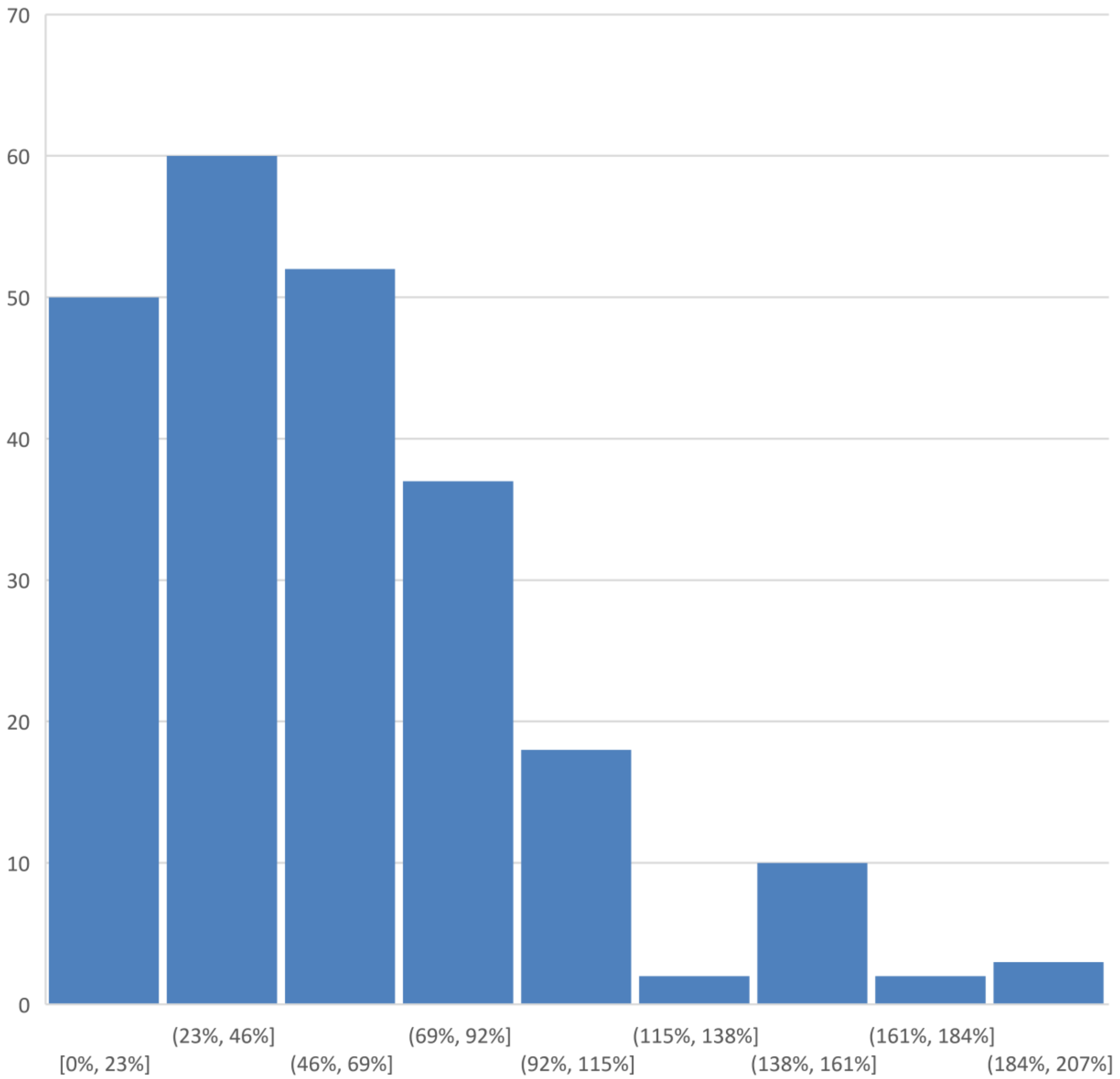

Supplementary figure S1. Variation of the EPC index. The variation coefficient (CV%) was calculated for the 4 biological replicates of the 26 barcodes monitored in the 9 plant co-inoculated plant genotypes (n=224). Conditions with 2 repeats displaying no reads were excluded of the analysis (n=10). Histogram of CV% is presented. Half of the samples displayed CV variations <50%.

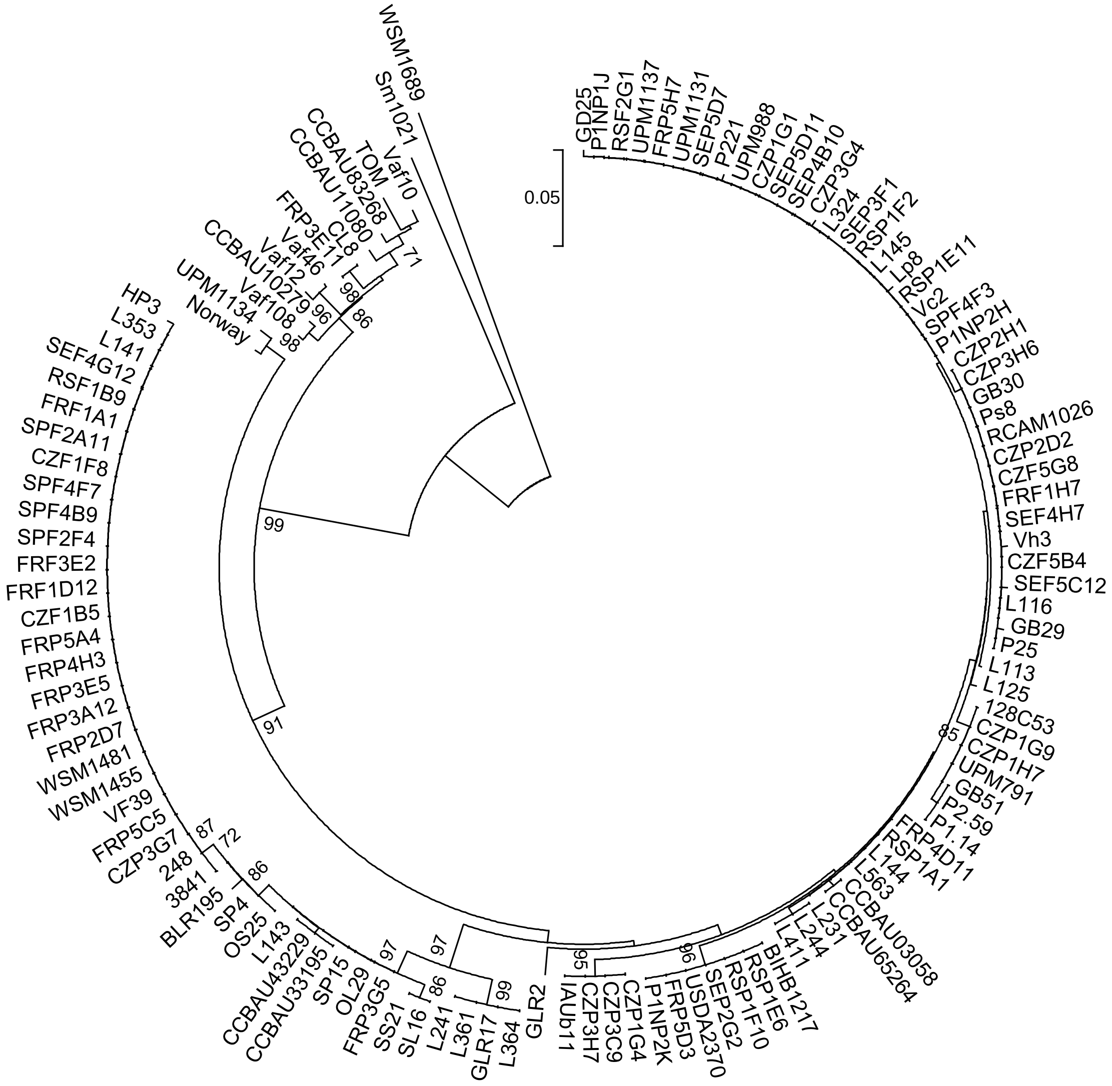

Supplementary figure S2: NodD309 barcode diversity among *Rhizobium leguminosarum* sv. *viciae.*

Phylogenetic tree of the nodD309 sequences present in the 121 genomes of *Rhizobium leguminosarum* sv. *viciae* selected for this study. WSM1689 (*R.leguminosarum sv. trifolii*) and *Sinorhizobium meliloti* 1021 have been used as outgroups.
